# Potential infliximab-induced Kounis syndrome in a patient with metastatic melanoma 

**DOI:** 10.5414/ALX02612E

**Published:** 2026-02-24

**Authors:** Corsin Seeli, Dimitri Patriki, Omar Hasan Ali, Ann-Kathrin Blumenröther, Marie-Charlotte Brüggen, Andrea Nolting*, Carole Guillet*

**Affiliations:** 1 Department of Dermatology, and; 2 Department of Cardiology, University Hospital Zurich, Switzerland; *These two authors contributed equally to this work.

**Keywords:** infliximab, monoclonal antibody hypersensitivity, acute coronary syndrome, Kounis syndrome, mast cell activation, immune-mediated reaction

## Abstract

Background: Kounis syndrome is a rare type of allergic or hypersensitivity-induced acute coronary syndrome. The release of numerous inflammatory mediators induces vasospasms and potential thrombosis leading to myocardial infarction. Case history: We present the case of a 65-year-old woman with metastatic melanoma who experienced chest tightness and dyspnea after her 3^rd^ infliximab infusion and who had transitory ST elevations. Suspecting allergic reactions and acute coronary syndrome she was simultaneously treated for both conditions with complete resolution of symptoms and electrocardiographic alternations without the need for further percutaneous coronary intervention. Conclusion: This case highlights the importance of considering Kounis syndrome as a potentially life-threatening complication during monoclonal antibody infusion, even in the absence of a prior allergic history and typical skin manifestations. Early recognition and treatment are crucial and spared our patient further invasive diagnostical and therapeutical interventions.

## Introduction 

Kounis syndrome (KS), also called allergic angina or allergic myocardial infarction, is an allergy- or hypersensitivity-induced acute coronary syndrome. First detailed in 1991 by Kounis [1], its pathophysiology involves mast cell and basophil activation during allergic responses [2]. This results in the rapid release of numerous inflammatory mediators, such as histamine, tryptase, chymases, leukotrienes, prostaglandins, and platelet-activating factor (PAF), that induce vasospasms, increase vascular permeability, and promote platelet activation as well as thrombosis. Myocardial and plaque-resident mast cells may contribute to plaque erosion or rupture via the secretion of proteolytic enzymes. Mediators such as tryptase can further destabilize thrombi, whereas leukotrienes and chymases act as potent vasoconstrictors [2]. 

To date, a broad spectrum of trigger factors for KS has been documented in the literature. Commonly implicated are non-steroidal anti-inflammatory drugs (NSAIDs), antibiotics, and contrast media, while environmental factors such as insect stings, certain foods, and stents have also been reported [3]. Moreover, the expanding use of biological agents – now indispensable in the management of oncological, immunological, and inflammatory disorders – has been accompanied by a notable increase in reported hypersensitivity reactions [4]. We share the case of a patient without allergic background who was receiving monoclonal antibody infusion with infliximab and developed acute chest pain and dyspnea. 

## Case history 

A 65-year-old woman with stage IV melanoma developed immune-mediated colitis while receiving nivolumab treatment. To manage this, infliximab was initiated (5 mg/kg), and the first 2 infusions were well tolerated. She had no known allergies or prior infusion-related events. 

**Figure 1:**
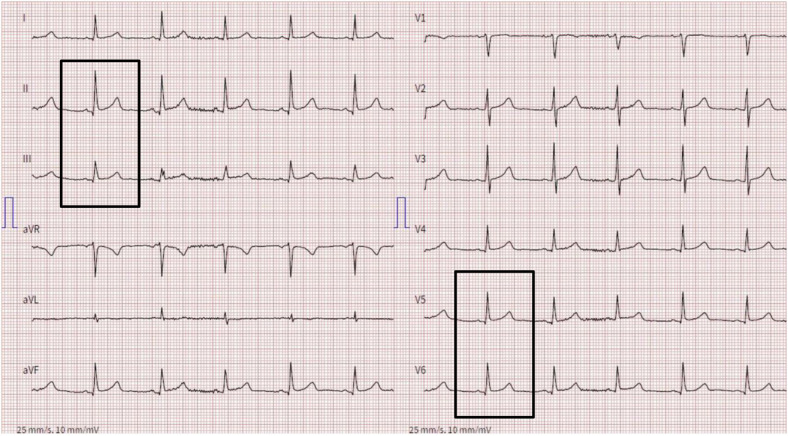
Electrocardiogram during the episode. Demonstrating ST-segment elevations (≥ 1 mm) in leads II, III, V5, and V6.

15 minutes into the 3^rd^ infliximab infusion, the patient experienced sudden onset of chest tightness and dyspnea. Her vital signs were initially normal, and she later presented with hypertension. Electrocardiography (ECG) revealed ST-segment elevations of uncertain significance (barely ≥ 1 mm) in leads II, III, and V5–6 (Figure 1), raising concern for acute coronary syndrome (ACS). Intravenous methylprednisolone (250 mg) and clemastine (2 mg) were administered promptly for presumed allergic reaction, as were aspirin and sublingual nitroglycerin for presumed ACS. This led to quick resolution of symptoms. 

Cardiac biomarkers (high-sensitive troponin T (hsTnT) and NT-proBNP) levels remained within normal limits. Echocardiography revealed a preserved left ventricular ejection fraction without regional wall motion abnormalities. The serum tryptase level measured 90 minutes after symptom onset rose from a baseline value of 7.65 to 12.3 µg/L, indicating systemic mast cell activation. 

**Figure 2:**
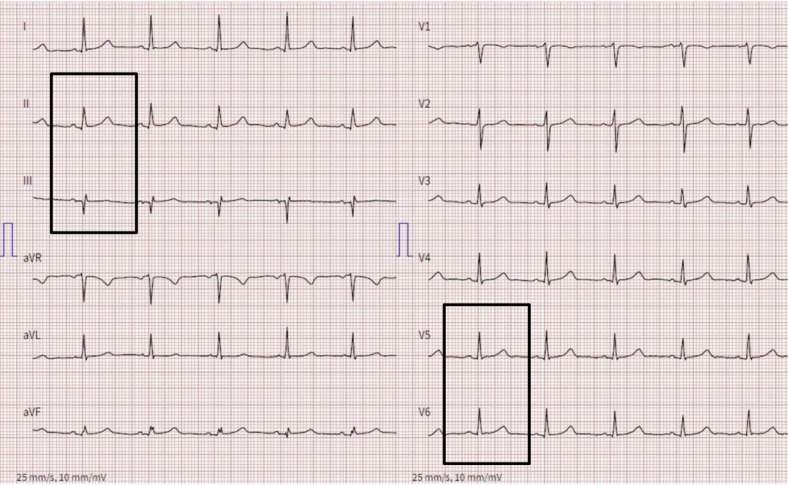
Electrocardiogram (ECG) recorded 2.5 hours after the onset of symptoms with tracings returned to baseline.

A follow-up ECG after 2.5 hours showed resolution of ST-segment changes (Figure 2). Owing to the presence of an alternative diagnosis that promptly responded to medical therapy, complete resolution of symptoms, regressive ECG changes, and non-relevant hsTNT changes, coronary angiography was not pursued. 

Allergy testing was performed 5 weeks later under ongoing hydrocortisone therapy (30 mg/day). An infliximab skin test was performed. Additionally, a skin test with polysorbate 80 was performed with polysorbate 80 1% skin prick testing (1 : 1) and intradermal testing (1 : 10, 1 : 100). All skin prick tests and intradermal tests yielded negative results. Additionally, anti-infliximab IgG (an anti-drug antibody) was negative. The patient had no prior history of cardiac events, did not smoke, and was treated for arterial hypertension. A prior echocardiogram revealed mild subaortic septal hypertrophy without outflow obstruction. 

This presentation – acute chest pain with transient ST elevations, elevated tryptase, and rapid improvement with antiallergic and vasodilatory treatment – suggests the diagnosis of type I KS. 

## Discussion 

KS can be categorized into three subtypes. Type I occurs without pre-existing coronary artery disease (CAD), where inflammatory mediators induce coronary spasm, leading to ischemia or angina. Type II may involve allergic inflammation triggering plaque rupture in existing CAD. Type III involves stent thrombosis. The clinical presentation of KS varies from mild chest pain to severe myocardial infarction with shock. Cardiac symptoms include angina, palpitations, and dyspnea. Allergic symptoms (rash, wheezing, hypotension) are often present. Diagnosis relies on patient history, especially concerning recent allergen exposure, ECG, biomarkers, and imaging. ECG often reveals ST-segment elevations or other ischemic changes. The described ST elevations in our patient’s ECG have been evaluated by a cardiologist. Based on the guidelines of the European Society of Cardiology of 2023, in women, new ST elevations in at least 2 contiguous leads ≥ 1 mm (and/ or ≥ 1.5 mm in V2 and V3) are considered as suggestive of acute coronary occlusion [5]. Echocardiography may reveal wall motion abnormalities; angiography may show spasm or thrombosis [2]. 

Treatment poses a challenge, as both the allergic and cardiac aspects must be addressed. Allergen discontinuation is essential. Standard ACS therapy is indicated, however, in type I KS, treating the allergic reaction alone may resolve the symptoms, as in this case. In more severe cases, percutaneous coronary intervention (PCI) may be necessary. Long-term management focuses on allergen avoidance [1]. 

Infliximab is a widely used anti-TNF agent. While ACS after infliximab administration has been reported, KS cases are exceedingly rare [6, 7]. However, it remains unclear whether this is solely due to its rarity or to underdiagnosis [8]. 

Diaz-Rodriguez et al. [7] reported a case of KS occurring a few minutes after the 3^rd^ administration of infliximab. In addition to cardiovascular symptoms, the described patient also presented with a maculopapular rash [7]. However, KS can occur without skin manifestations, and it is likely that cases are missed because of a lack of allergologic work-up thereafter [6, 9]. Typically, immediate-type reactions due to infliximab occur during or within 1 – 2 hours after completion of the infusion. According to a large registry, the most common symptoms of infliximab-related infusion reactions are pruritus (22.1%), flushing (9.9%), dyspnea (6.2%), chest discomfort (5.9%), hypertension (5.9%), myalgia (5.0%), nausea (4.7%), urticaria (4.7%), headache (4.0%), rash (3.4%), and dizziness (2.8%) [10]. Differentiating between various types of immediate infusion reactions is often challenging but certain features may provide important diagnostic clues: IgE-mediated reactions typically require prior sensitization. Symptoms such as wheezing and pronounced urticaria suggest significant histamine release and are indicative of either an IgE-mediated response or direct mast cell degranulation. An increase in serum tryptase levels supports the diagnosis of mast cell degranulation, whereas the presence of fever is more consistent with cytokine release [10]. Mast cell activation likely occurred via non-IgE mediated mechanisms, given the negative skin test results and concurrent corticosteroid use, which may have suppressed test reactivity. Inflammatory mediators released during mast cell degranulation may provoke coronary vasoconstriction and transient myocardial ischemia without necrosis and therefore normal cardiac biomarkers, as observed here [1]. Vasospasm could not be confirmed as angiography was not performed during the event. Immune checkpoint inhibitors can have cardiotoxic effects, which may present with similar symptoms. Distinguishing between hypersensitivity-related cardiac events and drug-induced cardiotoxicity has significant therapeutic implications [8]. Additionally, immune checkpoint inhibitors influence the regulation of the immune system, mainly via T cells. It is currently unclear whether they also increase the disposition towards hypersensitivity reactions. 

## Conclusion 

Our case highlights the importance of considering allergic myocardial ischemia in patients presenting with ACS-like symptoms during monoclonal antibody infusion, even in the absence of a prior allergic history and typical skin manifestations. Even though only minimal ECG changes occurred with appropriate symptoms, adequate therapy certainly prevented a more severe reaction and spared the patient further invasive diagnostics and therapeutic interventions. Clinicians should maintain a high index of suspicion as early recognition and prompt treatment are crucial. In this patient, infliximab was permanently discontinued. 

## Informed consent 

Written informed consent was obtained from the patient for publication of this case report, with the understanding that no identifiable information would be included. 

## Authors’ contributions 

Supervision: CG, PSG; Conception and design: CS, DB, CG, AKB; Data collection and investigation: CS, DB, AKB, OHA; Manuscript drafting: CS, DB, AN; Proof-reading: CS, DB, AN, AKB, OHA, PSG, CG. 

## Funding 

There is no funding to declare for all the authors. 

## Conflict of interest 

The authors declare that they have no conflict of interest.
